# Rationale for the Initiation, Outcomes, and Characteristics of Chemotherapy Following CDK4/6 Inhibitors in Breast Cancer: A Real-World Cohort Study

**DOI:** 10.3390/cancers16162894

**Published:** 2024-08-20

**Authors:** Miroslawa Püsküllüoğlu, Marek Ziobro, Joanna Lompart, Agnieszka Rudzińska, Tomasz Zemełka, Justyna Jaworska, Sebastian Ochenduszko, Aleksandra Grela-Wojewoda

**Affiliations:** 1 Department of Clinical Oncology, Maria Sklodowska-Curie National Research Institute of Oncology, Kraków Branch, 31-115 Kraków, Polandagnieszka.rudzinska@krakow.nio.gov.pl (A.R.); tomasz.zemelka@krakow.nio.gov.pl (T.Z.); aleksandra.grela-wojewoda@krakow.nio.gov.pl (A.G.-W.); 2 Hospital Universitario Doctor Peset, 46017 Valencia, Spain; sebaochenduszko@gmail.com

**Keywords:** luminal breast cancer, metastases, chemotherapy, CDK4/6 inhibitors, systemic treatment

## Abstract

**Simple Summary:**

This study investigated one of the treatment options for advanced breast cancer patients after they completed standard therapy that combines hormone therapy and specific targeted agents called cyclin-dependent kinase 4/6 inhibitors. We wanted to understand why in real-world patients start chemotherapy after finishing standard initial treatment, how they respond to it, and what factors might affect their outcomes. We found that chemotherapy was often used when patients faced dissemination to internal organs with the risk of further progression, but it provided limited benefits. We suggest that modern drugs recommended by guidelines before chemotherapy should be better reimbursed in Poland. This research could help doctors make better treatment decisions and improve future clinical trials.

**Abstract:**

The standard therapy for hormone-receptor-positive, human epidermal growth factor receptor 2-negative advanced breast cancer includes the use of cyclin-dependent kinase 4/6 inhibitors (CDK4/6i) with endocrine therapy. The optimal post-CDK4/6i treatment sequence is unclear. This cohort study evaluated the initiation, characteristics, and outcomes of chemotherapy following CDK4/6i-based treatment. Among the 227 patients who began CDK4/6i therapy, 114 completed it. Seventy-nine female patients received further treatment, including 55 receiving chemotherapy. The average age was 60.1 years. Post-CDK4/6i chemotherapy was typically (69.1%) first-line due to an impending visceral crisis. The median progression-free survival (mPFS) was 3.0 months (range 0.5–18.9), and the median overall survival (mOS) was 8.3 months (0.5–26.1). The median OS from the end of CDK4/6i treatment was 12.4 months (1.5–26.8). In univariate analysis, neither mPFS nor mOS was associated with age, tumor grade, receptor status, Ki67 status, time from diagnosis to CDK4/6i cessation, therapy line, or CDK4/6i type. Dose reduction occurred in 12 patients (21.8%), and chemotherapy was ceased due to adverse events in 8 patients (14.6%). Chemotherapy showed limited benefit regardless of the regimen. The role of chemotherapy may evolve with broader CDK4/6i use in adjuvant treatment.

## 1. Introduction

Breast cancer (BC) represents a growing health, social, and economic challenge worldwide, as reflected in statistical data. More than 2.3 million new cases of BC are diagnosed annually worldwide, constituting 11.6% of all newly diagnosed malignancies. Consequently, BC is one of the most commonly diagnosed cancers worldwide and the fifth leading cause of cancer-related death [1].

The median overall survival (mOS) for patients with advanced BC (ABC) is approximately 3 years, with a 5-year survival rate of 25% [2,3]. The prognosis largely depends on the specific subtype of BC. On the basis of the gene expression data, five biological subtypes were identified: luminal A, luminal B, human epidermal growth factor receptor 2 (HER2)-positive, basal, and “normal-like” BC. In clinical practice, these criteria are replaced with equivalents on the basis of routinely assessed pathomorphological features of invasive BC, such as estrogen/progesterone receptor (ER/PR) status, HER2 status, and the Ki67 proliferation index. Approximately 75% of invasive BCs express hormone receptor (HR) and lack HER2 overexpression/amplification. Therefore, the luminal HER2-negative subtype is the most commonly recognized biological subtype of BC [4,5,6].

Significant progress has been made in the systemic therapy of patients with HR-positive HER2-negative ABCs in recent years. The introduction of a new group of drugs, Cyclin-Dependent Kinase 4/6 inhibitors (CDK4/6i), has been a major change. CDKs are a critical subgroup of protein kinases involved in regulating the cell cycle, transcription, and gene expression [6,7]. Among the 21 CDK genes in the human genome, CDK4/6 inhibitors (CDK4/6i) have become a cornerstone of treatment for luminal HER2-negative BC. Normally, CDK4/6 forms a complex with cyclin D, phosphorylating the retinoblastoma (RB) protein and releasing the E2F transcription factor, which drives the cell cycle from G1 to S phase, leading to DNA replication. In HR+ BC, estrogen activates the ER signaling pathway, increasing cyclin D and CDK4/6 expression, resulting in uncontrolled cell proliferation. CDK4/6 inhibitors prevent RB phosphorylation and E2F release, thereby arresting cell cycle progression [8]. Although primarily used in combination with endocrine therapy, CDK4/6i have demonstrated efficacy across different cancer subtypes. Notably, CDK4/6i combined with anti-HER2 therapies have shown promise in treating HER2-positive BC, offering an alternative for patients unable or unwilling to receive chemotherapy. Despite the progress, the exploration of CDK inhibitors as monotherapy and in various combinations remains ongoing [6,7].

CDK4/6i registration trial results confirmed the efficacy of three drugs from this group (abemaciclib, palbociclib, and ribociclib) in combination with endocrine therapy (ET) in both the first and second lines of systemic palliative treatment in luminal HER2-negative ABC. Combination therapy allows for extending the median progression-free survival (mPFS) and mOS and maintaining or improving the health-related quality of life (HRQoL) of patients [9,10,11,12,13,14,15]. An important problem remains the sequence of palliative therapies in subsequent lines after progression on CDK4/6i in combination with hormone therapy. Nevertheless, guidelines advocate a tailored approach to target identification in HR+/HER2-negative ABC, focusing on tumor and patient factors to optimize treatment selection and guide clinicians in sequencing therapies [2,16,17,18].

In SOLAR-1, alpelisib significantly improved the mPFS when combined with fulvestrant in patients with *phosphatidylinositol*-4,5-*bisphosphate* 3-*kinase catalytic subunit alpha (PIK3CA)*-mutated, aromatase inhibitor-resistant BC [19]. A large phase II BYLieve study revealed the effectiveness of alpelisib after prior use of CDK4/6i [20]. An alternative treatment pathway may be the use of exemestane (a steroidal aromatase inhibitor) in combination with everolimus. According to the results of the BOLERO-2 study, such treatment was associated with a longer mPFS than was exemestane alone [21]. In the EMERALD phase III trial, the effectiveness of elacestrant, an oral selective estrogen receptor degrader, was assessed in comparison to standard endocrine therapy among individuals who had previously progressed on other ETs. The results revealed a significant improvement in mPFS for all participants, with notably greater benefits observed among those with an *estrogen receptor 1* gene mutation. The continuation of therapy based on CDK4/6i following progression represents an approach of unconfirmed value. Two large randomized phase II trials, MAINTAIN and PACE, dedicated to this treatment strategy have provided conflicting conclusions [22,23].

Patients harboring germline pathogenic *BReast CAncer* gene (*BRCA1/2*) mutations may benefit from monotherapy with the poly ADP-ribose polymerase (PARP) inhibitors olaparib or talazoparib [24,25].

In the DESTINY-Breast04 phase III trial, the effectiveness of trastuzumab deruxtecan (T-DXd) was superior to that of a physician’s choice of chemotherapy in patients diagnosed with HER2-low metastatic BC who had undergone one or two prior lines of chemotherapy [26]. According to the phase III trial TROPiCS-02 findings, patients with HR-positive/HER2-negative metastatic BC who have undergone at least two prior lines of chemotherapy should be treated with sacituzumab govitecan (SG) [27]. Patients who exhaust the possibility of ET and other available treatment options and those with signs of visceral crisis (VC) can require chemotherapy on the basis of active regimens, similar to patients with the triple-negative BC subtype [2,18].

The aim of this retrospective cohort study was to assess the reasons for chemotherapy initiation, its characteristics, and outcomes in hormonal-resistant HER2-negative BC patients who completed their first- or second-line treatment with CDK4/6i.

## 2. Materials and Methods

### 2.1. Patients

Patients who were diagnosed with ABC and treated at the Maria Sklodowska-Curie National Research Institute of Oncology, Branch Krakow, who completed their treatment with CDK4/6i + ET, either due to disease progression or treatment discontinuation for any reason between September 2019 and December 2023, were included in the study. The inclusion criteria were individuals aged 18 years or older, demonstrating ER or PR levels ≥ 1%, and exhibiting a HER2-negative status. The exclusion criterion was patients who had received CDK4/6i treatment for early-stage disease. There were no limitations regarding patient sex, the type or line (in the palliative setting) of CDK4/6i, or the type or line of chemotherapy regimens used after CDK4/6i treatment.

### 2.2. Data Collection

We compiled patient demographic data, including age, sex, menopausal status, and histopathological details (such as histological subtype; ER, PR, and HER2 status; Ki-67 proliferation index; presence of ductal carcinoma in situ (DCIS); tumor grade; presence of lymphovascular invasion (LV); and various breast cancer molecular subtypes). Additionally, we recorded the date of initial diagnosis; date of metastasis; site of metastasis; previous systemic treatment administered; type, line, and median duration of CDK4/6i therapy; and survival status. These data were collected prospectively in accordance with the requirements of the reimbursement program in Poland.

Furthermore, we retrospectively gathered information on the rationale for initiating post-CDK4/6i therapies and patient characteristics (including the type of regimens, number of cycles, doses, safety concerns, dose reductions, cessation of chemotherapy owing to side effects, and radiological assessments) from patient records or the hospital registry system. For patients who did not receive chemotherapy, we collected information on the reasons for not initiating chemotherapy at any point.

### 2.3. Study Objectives

The primary objective of this investigation was to determine the mPFS and mOS associated with the first chemotherapy treatment following CDK4/6i administration. The secondary objectives included assessing the safety of chemotherapy treatment and overall response rates (ORRs). Additionally, we evaluated the differences in mPFS between patients who received at least one line of chemotherapy and those who did not receive chemotherapy after CDK4/6i treatment until death.

### 2.4. Ethical Considerations

This study received approval from the Ethical Committee at the Maria Sklodowska-Curie National Research Institute of Oncology, Branch Warsaw, Poland (20/2024, dated 22 February 2024). Given the retrospective design of the study, the Ethical Committee granted an exemption from providing informed consent. All procedures and protocols adhered to the pertinent guidelines and regulations.

### 2.5. Statistical Analysis

The means, standard deviations, medians, quartiles, and ranges of the quantitative variables are shown. For qualitative variables, absolute and relative frequencies (N and %) are reported. The Mann-Whitney test was used for comparisons of quantitative variables between two groups, whereas the Kruskal-Wallis test (followed by the post hoc Dunn test) was used for comparisons of three or more groups. Spearman’s correlation coefficient was used to assess the correlation between two quantitative variables. Univariate Cox regression (proportional hazards model) was employed to model the impact of potential predictors on a time to event. HRs (hazard ratios), in addition to 95% confidence intervals, are presented.

The significance level was set to 0.05. All the analyses were conducted in R software, version 4.3.3.

## 3. Results

### 3.1. Patient Selection and Characteristics

**Figure 1:**
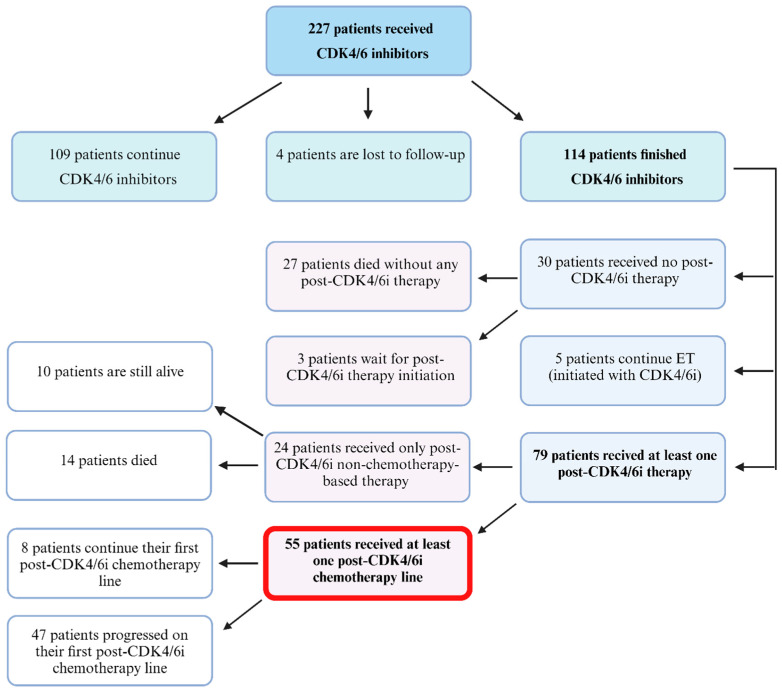
Flow chart of the study. Abbreviations: CDK4/6i, cyclin-dependent kinase 4/6 inhibitors.

Among patients who finished ET+CDK4/6i therapy (due to any reason), 79 patients received at least one subsequent treatment line, including 55 patients who received at least one cycle of chemotherapy during their post-CDK4/6i treatment period. Figure 1 shows the patients who were selected for analysis.

All the patients who received at least one line of post-CDK4/6i chemotherapy were females. The mean age of the patients at the time of chemotherapy initiation was 60.17 years (SD 12.57), the median age was 59.25 years (quartiles: 52.13–70.49), and the age range was 32.53–81.55 years. The population characteristics are presented in Table 1.

### 3.2. Chemotherapy Regimen Characteristics and Rationale for Initiation

The characteristics of the post-CDK4/6i chemotherapy regimens are presented in Table 2.

Among these 55 patients, 2 received alpelisib + fulvestrant, and none received PARP inhibitors in any post-CDK4/6i lines of treatment (only one patient out of 12 tested had a BRCA2 mutation).

Among the reasons for chemotherapy initiation, the most common were VC or impending VC (22 patients, 40.00%); however, when patients’ histories were assessed, only 15 patients (27.27%) had VC or impending VC according to the ABC 5 criteria [2]. The other patients (7; 12.73%) experienced rapid progression. Further reasons were as follows: physician’s decision (19 patients; 34.55%), no other ET treatment options available (13 patients; 23.64%), or other reasons (1 patient; 1.82%).

### 3.3. Factors Influencing Post-CDK4/6i Chemotherapy Initiation

The mean time from the initiation of CDK4/6i therapy to chemotherapy initiation was 3.15 months (SD 3.66), with a median of 1.38 months (quartiles: 0.67–4.81) and a range of 0.03–14.26 months. The logistic regression models revealed that none of the analyzed features were significant predictors of the likelihood of receiving chemotherapy (all *p* > 0.05): ER and PR expression, HER2 expression, tumor grade, Ki67 index, patient age, CDK4/6i treatment duration, time from diagnosis to CDK4/6i treatment end, time from metastatic disease diagnosis to CDK4/6i treatment end, line of treatment with CDK4/6i, stage at the time of diagnosis, presence of visceral metastases, type of CDK4/6i used, and time from CDK4/6i therapy end to chemotherapy initiation.

In addition, none of the abovementioned factors influenced the line of treatment in which first post-CDK4/6i chemotherapy was applied, apart from (1) the time from metastatic disease diagnosis to the end of CDK4/6i therapy (the longer the time, the later the line at which chemotherapy was initiated with a Spearman’s correlation coefficient of 0.34 and *p* = 0.011), and (2) the type of CDK4/6i applied (the line at which chemotherapy was initiated was significantly later in patients receiving palbociclib than in those receiving ribociclib, *p* = 0.009).

### 3.4. Chemotherapy Outcomes and Influencing Factors

**Figure 2:**
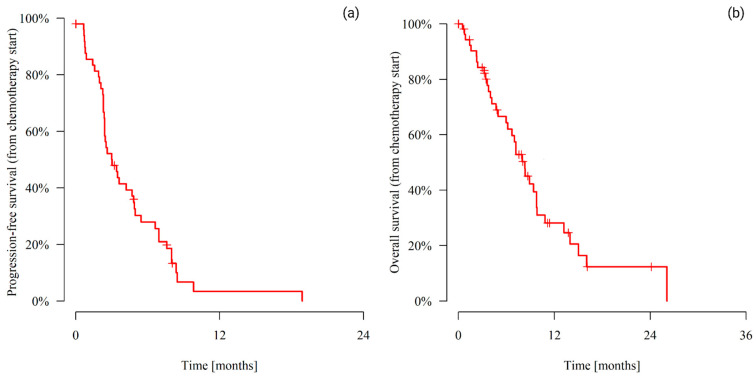
Median progression-free survival (**a**) and overall survival (**b**).

The median PFS for the first chemotherapy line initiated after CDK4/6i therapy was 3.02 months (range 0.53–18.89), whereas the median OS (from chemotherapy initiation) was 8.31 months (range 0.53–26.09). Figure 2 and Table 3 present the mPFS and mOS.

Neither mPFS nor mOS (from chemotherapy initiation) was related to the following factors: patient age, tumor grade, ER (%), PR (%), HER-2 status, Ki67 status, presence of visceral metastases, previous application of chemotherapy in any setting, time from diagnosis to CDK4/6i therapy end, time from diagnosis of metastatic disease to CDK4/6i therapy end, length of CDK4/6i therapy, line of palliative therapy in which CDK4/6i was administered (first line, second line), or administration of any type of chemotherapy before CDK4/6i or type of CDK4/6i was used.

The Cox proportional hazards model revealed that patients initially diagnosed with nonmetastatic disease had a 2.639-fold greater risk of progression or death at any time (HR = 2.639) than patients diagnosed with de novo metastasis did. One-line postponement of post-CDK4/6i chemotherapy increased the likelihood of death at any time by 2.023 times (HR = 2.023), whereas the likelihood of progression or death at any time increased by 92.7% (HR = 1.927). The median OS (post-CDK4/6i OS) from the end of CDK4/6i treatment to patient death was 12.39 months (range 1.54–26.81). Using platinum-based treatment increased the likelihood of death at any time by 2.377 times (HR = 2.377).

Patients’ radiological responses were assessed as per the Response Evaluation Criteria in Solid Tumors (RECIST) 1.1. In total, 3 patients (5.45%) experienced partial response as their best response; 27 patients (49.09%) had stable disease; 18 patients (32.73%) had radiologically progressive disease (PD) or clinical progression; and other patients still awaited their first radiological assessment or had an unknown response.

### 3.5. Chemotherapy Safety

The majority of the patients (44; 80.00%; including 5 patients still on treatment) did not experience a grade > 2 adverse event (AE) according to the Common Terminology Criteria for Adverse Events v. 5.0 during chemotherapy. Among the 11 patients who experienced grade >2 toxicity, 3 had hematological toxicity, 3 had infections, 2 had allergic reactions, 1 had gastrointestinal disease, 1 had fatigue, and 1 had neurological adverse effects.

Dose reduction was required in 12 patients (21.82%), chemotherapy was delayed in 13 patients (23.64%), and chemotherapy was ceased due to AEs in 8 patients (14.55%). The reasons for ending chemotherapy were disease progression (27 patients, 49.09%), completing the preplanned number of cycles (10 patients; 18.18%), AEs (8 patients, 14.55%), physician decision cessation due to maximal clinical benefit (2 patients; 3.64%), and infection unrelated to chemotherapy (1 patient; 1.82%).

### 3.6. Factors Responsible for Post-CDK4/6i Chemotherapy Noninitiation and Group Comparisons

Among 14 patients (see Figure 1) who did not receive chemotherapy as a part of post-CDK4/6i treatment but received at least 1 subsequent line of therapy and died (thus, they were not candidates for chemotherapy in the future), 13 (92.86%) patients managed to receive only one line of treatment. The reasons for not receiving further lines of chemotherapy were progression of the disease causing patient deterioration or patient death due to disease progression) in 12 patients (85.71%) and patient comorbidities in 1 patient (7.14%). Only one patient received three lines of nonchemotherapy-based post-CDK4/6i treatment. Table 4 shows the OS data compared for patients who received chemotherapy and those who did not receive it until death.

## 4. Discussion

Combination therapy with CDK4/6i and ET is the treatment of choice for patients with luminal HER2-negative ABC without VC features for postmenopausal and premenopausal patients (provided luteinizing hormone-releasing hormone (LHRH) agonists are administered or ovariectomy is performed) [2,18]. International guidelines do not specify a standard category of first-line treatment for subsequent therapy following a CDK4/6i-based regimen [2,18].

In Poland, CDK4/6i have been reimbursed since September 2019 (abemaciclib since September 2020). Currently, alpelisib in PIK3CA-mutated patients in combination with fulvestrant (since November 2022) as well as olaparib (since November 2023) and talazoparib (November 2022) in BRCA1/2-mutated individuals are reimbursed after progression on CDK4/6i therapies. Neither everolimus, elacestrant, CDK4/6i after progression on CDK4/6i, capivasertib, antibody-drug conjugates such as SG, nor T-DXd are reimbursed options in this population [28,29].

According to our data, approximately half of the patients received chemotherapy due to the appropriate cause being present or impending VC (27.27%) or the absence of alternative endocrine therapy options (23.64%). This proportion could be reduced by introducing new treatment modalities, which have been partially achieved with the recent reimbursement of alpelisib or PARP inhibitors in Poland. Unfortunately, numerous patients started chemotherapy, although other options were available, including 12.73% with rapid progression (and without VC) and as many as 34.55% due to physician decisions. The outcomes emphasize the need to understand the underlying reasons for such practices. Are they due to deficiencies in substantive training, issues in patient communication, or succumbing to pressure to administer chemotherapy as a “stronger treatment”? Such results could guide the retraining of oncologists so that these situations do not automatically warrant chemotherapy initiation. The detrimental preference for chemotherapy over hormonal agents, even in earlier lines of treatment for hormone receptor-positive HER2-negative ABC, has been emphasized in numerous publications [30]. On the other hand, other studies focusing on the post-CDK4/6i setting suggest that the relatively shorter median duration of CDK4/6i treatment among patients who received chemotherapy than among those who received endocrine therapy indicates that this subgroup might have a less favorable prognosis, potentially influencing the mPFS in the chemotherapy group (selection bias) [31]. Our nonchemotherapy group was very small and had very poor outcomes (Table 4); however, the duration from the diagnosis of metastatic disease to the completion of CDK4/6i therapy correlated with the later initiation of chemotherapy. Presumably, physicians observed that these women progressed more slowly and believed that they had time to decide on commencing chemotherapy, which appears to be a logical course of action. Other studies reported similar phenomena [32].

Even if more new treatment modalities were available for patients in our cohort, some patients would still require chemotherapy due to the heterogeneity of the ABC population. Specifically, certain subgroups may not meet the criteria for newer drug approvals or reimbursement or may present with aggressive disease features, such as visceral crisis, where chemotherapy remains a viable option [33]. Data regarding the efficacy and characteristics of chemotherapy following CDK4/6 inhibitors in luminal breast cancer in both clinical trials and real-world data (RWD) are limited (Table 5).

Numerically, our results revealed similar or poorer chemotherapy mPFS or mOS than did clinical trial outcomes (Table 5); however, some RWD concerning unselected populations progressing on CDK4/6i showed similar mPFSs of 4 months, without any statistically significant differences between hormonal therapy-based or everolimus plus hormonal therapy-based regimens [39]. In addition, Alghanmi et al. reported no notable difference in mPFS concerning the duration of first-line therapy or other clinicopathological factors [39]. Such correlations were suggested for RWD regarding post-CDK4/6i everolimus + hormonal therapy [42]. In the small cohort presented by Costa et al., chemotherapy was the preferred post-CDK4/6i regimen [40]. In the study by Xi et al., patients also received chemotherapy as the most common first-choice with mPFS: not determined, 4.7 months, and 4.1 months after the initiation of first-line palbociclib, second-line, and subsequent-line palbociclib, respectively (*p* = 0.56). In our study, the efficacy of chemotherapy (mPFS and mOS) was also associated with the CDK4/6i palliative line of treatment (without influencing OS from the end of CDK4/6i therapy to death). Furthermore, the selection of chemotherapy varied, as nab-paclitaxel or eribulin was not an option in our cohort [37].

Our findings suggest that the use of platinum increases the risk of death at any time by 2.377 times, which can be attributed to our local policy of administering platinum compounds to patients experiencing hepatic VC. This practice has also been documented by other research groups [43]. However, some studies, including ours, suggest better outcomes with capecitabine than with other regimens and do not show the superiority of any chemotherapy regimens [35].

Regardless of the type of therapy received, 24.77% of our patients did not receive any line of treatment subsequent to CDK4/6i therapy (Figure 1). In the study by Xi et al., this proportion was similar (21.80%) [37]. However, it is possible that some of these patients had contraindications to chemotherapy or refused it, while they could still start targeted treatment that had not been reimbursed at that time.

Individuals initially diagnosed with nonmetastatic disease face a greater likelihood of progression or mortality at any point than do those with de novo metastasis. This observation has been documented in prior research and may stem from their therapy-naïve status or reduced resistance to systemic treatments [44].

The significant delay in initiating chemotherapy among patients receiving palbociclib compared with those receiving ribociclib can be attributed to the lack of evidence showing an influence on mOS with palbociclib. However, available real-world data encourage the use of palbociclib as a promising and effective option, particularly in older patients (theoretically considered slower-progressing populations) [45,46]. Ultimately, in our study, the type of CDK4/6i did not impact the mPFS or mOS.

### Study Limitations

This study is subject to several limitations, primarily stemming from its single-center design and the relatively small and heterogeneous sample size. Notably, 61% of the patients were receiving third-line palliative chemotherapy, and a total of 47% received chemotherapy despite the availability of other options, which may impact the generalizability of the findings. Although the period for patient initiation of CDK4/6i therapy spans nearly four years, a considerable number of patients remain on treatment. Potentially extending the observation period would significantly increase the size of the study cohort. Next, the observational nature of the study can be a source of bias, such as selection bias, the impact of confounding variables on outcomes, information bias stemming from inaccurate data collection, survivorship bias, and challenges in establishing causal relationships without randomization [47]. In the present study, performing a multivariate analysis was technically not possible. A comparison between endocrine therapy and chemotherapy in terms of efficacy was not possible owing to the heterogeneity of the patients and the possible presence of selection bias. Due to inadequate drug reimbursement during much of the observational period, alternative treatment options, which are currently considered the standard of care or are used in the post-CDK4/6i setting, were not widely accessible. Consequently, our study population’s OS outcomes may not be representative of patients treated in countries with more robust reimbursement systems [48]. This renders our dataset particularly unique, providing insights into chemotherapy OS and post-CDK4/6i outcomes without the confounding influence of alternative (more effective) treatment options. Lack of drug reimbursement and limited access to genetic counseling can only partially explain low *BRCA1/2* mutation testing among the studied population.

As CDK4/6i transit to adjuvant treatment is ongoing, the landscape of palliative care in the HR-positive, HER2-negative BC population remains uncertain. Additionally, the role of chemotherapy in this clinical scenario is not only incompletely understood but also needs to be redefined.

## 5. Conclusions

Current guidelines advocate for a comprehensive approach to target identification, tailored to both tumor characteristics and patient-specific factors in advanced HR+/HER2-negative breast cancer. This strategy aims to ensure the selection of the most effective treatment within established guideline options and to assist clinicians in determining the optimal treatment sequence [2,16,17,18]. However, the most effective treatment sequence following CDK4/6i progression has not been identified. The majority of patients in our study were treated with post-CDK4/6i chemotherapy within a shorter timeframe. The most common reason for commencing chemotherapy was impending or existing VC. Chemotherapy regimens, including both single-agent and combination therapies, vary, with the choice of regimen depending on previously utilized lines of treatment. Regardless of the regimen employed, a previous response to CDK4/6i or the location of the metastases chemotherapy demonstrated restricted efficacy. Our findings revealed similar or inferior numerical outcomes for mPFS or mOS compared to those reported in clinical trials or RWD (Table 5). Alternative therapeutic modalities exist in this scenario and warrant consideration before resorting to chemotherapy. Studies aimed at understanding the reasons why oncologists initiate chemotherapy before more effective options and planning adequate training based on these results should be conducted. Improved drug reimbursement in Poland and appropriate tumor characterization are also necessary to adhere to current guidelines [2,16,17,18]. Currently, more new options are being tested [48], and there is growing knowledge about the molecular characterization of this BC group [49,50]. With the expanded use of CDK4/6i in adjuvant treatment, the role of chemotherapy in this context may undergo transformation.

## Figures and Tables

**Table 1:** Population of patients who received post-CDK4/6i chemotherapy.

Parameter		Total (N = 55)
Grade	G1	2 (3.64%)
	G2	32 (58.18%)
	G3	6 (10.91%)
	Unknown	15 (27.27%)
Estrogen Receptor (%)	Mean (SD)	86.46 (20.37)
	Range	20–100
Progesterone Receptor (%)	Mean (SD)	62.15 (32.62)
	Range	1–100
HER2 Immunohistochemistry	0	31 (56.36%)
	1	11 (20.00%)
	2 *	13 (23.64%)
Ki67 (%)	Mean (SD)	27.68 (13.12)
	Range	10–60
Stage at diagnosis	IV	14 (25.45%)
	I–III	41 (74.55%)
Visceral metastases	No	23 (41.82%)
	Yes	32 (58.18%)
CNS metastases	No	53 (96.36%)
	Yes	2 (3.64%)
Line of palliative therapy for CDK4/6i	1st line	30 (54.55%)
	2nd line **	25 (45.45%)
Reason for ending CDK4/6i therapy	Toxicity	2 (3.64%)
	Disease progression	47 (85.45%)
	Patient’s decision	1 (1.82%)
	Unknown/multiple	5 (9.09%)
CDK4/6i used	Abemaciclib	8 (14.55%)
	Palbociclib	24 (43.64%)
	Ribociclib	23 (41.82%)
Any chemotherapy in patients history used before CDK4/6i	No	23 (41.82%)
	Yes	32 (58.18%)
Line of palliative treatment chemotherapy was applied for the first time ***	2nd line	13 (23.64%)
	3rd line	34 (61.82%)
	4th line	7 (12.73%)
	5th line	1 (1.82%)
Line of treatment chemotherapy was applied for the first time following CDK4/6i	1st line	38 (69.09%)
	2nd line	15 (27.27%)
	3rd line	2 (3.64%)
Number of post-CDK4/6i chemotherapy lines received	1 line	17 (30.91%)
	2 lines	15 (27.27%)
	3 lines	3 (5.45%)
	At least 1 line ****	14 (25.45%)
	At least 2 lines ****	5 (9.09%)
	At least 3 lines ****	1 (1.82%)

* Fluorescence in situ hybridization negative results. ** Among these 25 patients, 8 received palliative first-line chemotherapy, and the rest received palliative hormonal treatment as a first-line treatment. *** For post-CDK4/6i chemotherapy. **** Patients who are still alive and may receive further chemotherapy lines. Abbreviations: CDK4/6i, cyclin-dependent kinase 4/6 inhibitors; CNS, central nervous system; HER2, human epidermal growth factor receptor 2.

**Table 2:** Characteristics of chemotherapy following CDK4/6 inhibitors in breast cancer.

Parameter		Total (N = 55)
Chemotherapy type *	Taxane-based	17 (30.91%)
	Anthracycline-based	16 (29.09%)
	Platin-based	13 (23.64%)
	Capecitabine	9 (16.36%)
	Navelbine	3 (5.45%)
	Gemcitabine	1 (1.82%)
	Monotherapy	26 (47.27%)
	Combined therapy	29 (52.73%)

* Maybe more than one possible combination therapy was applied.

**Table 3:** Median progression-free survival and overall survival.

Patients	Events	Time (from Chemotherapy Start)			
		6 Months	12 Months	18 Months	Median (Months)
		Median progression-free survival			
47	44	27.90%	3.32%	3.32%	3.02
		Median overall survival			
36	36	64.31%	28.14%	12.31%	8.31

**Table 4:** Overall survival of patients who received post-CDK4/6i systemic treatment.

Group	Patients	Events	Overall Survival				*p*
			6 Months	12 Months	18 Months	Median (Months)	
Chemotherapy	55	36	74.96%	52.64%	20.01%	12.39	*p* < 0.001
No chemotherapy *	14	14	42.86%	7.14%	>max obs.	4.24	

p-LR (log-rank) test. > max obs.—extends the longest observation time in this group. * In postCDK4/6i setting.

**Table 5:** Original studies regarding chemotherapy after CDK4/6 inhibitors in advanced breast cancer [31,32,34,35,36,37,38].

Ref.	Year	N	Type of Study	Prior CDK4/6i Treatment	CTH Regimen Characteristics	Outcomes (for CTH Arm)	Comments
[34]	2022	1210, of which 249 received CTH	Retrospective cohort study–nationwide database	palbociclib n = 1067 abemaciclib n = 56 ribociclib n = 87 ET agents: anastrozole n = 59 exemestane n = 28 fulvestrant n = 366 letrozole n = 745 tamoxifen n = 12	The study lacks information on specific CTH regimens.	rwPFS 3.71 mo	Continuation of CDK4/6i was associated with a significantly improved rwPFS compared to CTH. Treatment with fulvestrant monotherapy or everolimus was not observed to have statistically significant benefits in rwPFS compared to CTH. Treatment with everolimus was associated with improved OS compared to CTH, but fulvestrant was not.
[31]	2023	609, of which 434 received CTH	Retrospective cohort study	The study lacks information on which CDK4/6i was used. Group A: 1st line CDK4/6i median duration 10 mo Group B: 2nd line CDK4/6i median duration 9 mo Group C: ≥3rd line CDK4/6i median duration 5 mo	1st line CDK4/6i → CTH n = 126; taxane n = 48 capecitabine n = 37 carboplatin + taxane n = 17 anthracycline + cyclophosphamide n = 12 gemcitabine n = 5 cisplatin + gemcitabine n = 4 taxane + cyclophosphamide n = 3 2nd line CDK4/6i →CTH n = 110; taxane n = 32 capecitabine n = 44 carboplatin + taxane n = 9 anthracycline + cyclophosphamide n = 4 gemcitabine n = 7 cisplatin + gemcitabine n = 10 vinorelbine n = 4 ≥3rd line CDK4/6i → CTH n = 198; taxane n = 43 capecitabine n = 50 carboplatin + taxane n = 23 anthracycline + cyclophosphamide n = 6 gemcitabine n = 24 cisplatin + gemcitabine n = 15 vinorelbine n = 22 eribulin n = 10 ixabepilone n = 3	mPFS group A: ET 9.5 mo; CTH 5.3 mo group B: ET 6.7 mo; CTH 5.7 mo group C: ET 5.3 mo; CTH 4.0	Most frequently used CTH were capecitabine and taxanes. No significant difference was found comparing mPFS between ET and CTH groups. CTH was preferred to ET in patients, whose disease progressed shortly after starting CDK4/6i. The shorter median duration of CDK4/6i in patients who received CT compared to ET suggested that this group might have a relatively poor prognosis, which could affect the mPFS in CTH group.
[35]	2023	305, of which 80 received CTH	Retrospective cohort study RWD	palbociclib + letrozole	capecitabine n = 47 taxane n = 28 anthracycline n = 3 other CTH n = 2	mPFS: capecitabine 7.4 mo other CTH (taxane, anthracycline, other CTH) 4.8 mo mOS: capecitabine 42.3 mo other CTH (taxane, anthracycline, other CTH) 23.1 mo	In visceral organ disease progression, capecitabine and cytotoxic CTH had significantly longer mPFS in comparison to fulvestrant. In patients with bone metastasis alone, capecitabine had superior PFS to other second-line regimens. The OS did not differ according to the second-line treatment
[36]	2023	543, of which 249 received CTH	Randomized controlled phase 3 trial	CDK4/6i + ET in the CTH arm: 1st line n = 101 2nd line n = 56 3rd line n = 49 ≥4th line n = 62	CTH arm: eribulin n = 130 vinorelbine n = 63 gemcitabine n = 56 capecitabine n = 22	mOS 11.2 mo ORR n = 38 Clinical benefit rate n = 60	All patients received previous CTH in the metastatic setting. SG demonstrated both improved PFS and OS over CTH in the endocrine-resistant, post-CDK4/6i setting. More patients in the SG arm compared to the CTH arm experienced grade 3 adverse events.
[32]	2018	525, of which 193 received CTH	Retrospective cohort study	CDK4/6i + ET	capecitabine n = 85 paclitaxel n = 53 gemcitabine n = 24 doxorubicin based n = 15 eribulin n = 12 other n = 4	No significant endpoints	Patients who transitioned from a CDK4/6i based line to CTH (vs. endocrine, everolimus, or subsequent CDK4/6i) were more likely to have recurrent rapidly progressing disease and were significantly less likely to have the prior CDK4/6i-based line in combination with an AI.
[37]	2019	200, of which 70 received CTH	Retrospective cohort study	palbociclib with: letrozole n = 147 fulvestrant n = 50 anastrozole n = 2 tamoxifen n = 1	capecitabine n = 21 eribulin n = 16 paclitaxel albumin-bound n = 15 other n = 18	mPFS 4.2 mo mPFS following progression on: 1st line palbociclib not reached, 2nd line palbociclib 4.7 mo; ≥3rd line palbociclib 4.1 mo	The mPFS was similar among patients on capecitabine, eribulin and paclitaxel. The study included 6 patients with HER2-positive tumors.
[38]	2021	59, of which 32 received CTH	Retrospective cohort study	PALOMA-2 palbociclib + letrozole PALOMA-3 palbociclib + fulvestrant	PALOMA-2 First line: paclitaxel + bevacizumab n = 2 paclitaxel n = 1 TS n = 1 Second line capecitabine n = 2 capecitabine + fulvestrant n = 1 docetaxel n = 1 eribulin n = 1 PALOMA-3 First line paclitaxel + bevacizumab n = 4 capecitabine n = 1 cyclophosphamide + doxorubicin n = 1 eribulin n = 1 Second line paclitaxel + bevacizumab n = 4 eribulin n = 4 TS-1 n = 3 cyclophosphamide + epirubicin n = 2 capecitabine n = 2 paclitaxel n = 1	Duration of subsequent therapy: PALOMA 2 First line 6.4 mo Second line 2.4 mo PALOMA-3 First line 3.8 mo Second line 5.8 mo	Compared with patients in PALOMA-2, CTH was used more frequently in PALOMA-3. The efficacy of the implemented CTH which the patients received after palbociclib progression was not assessed.
[39]	2023	59	Retrospective cohort study	palbociclib with AI	The study lacks information on specific CTH regimens.	mPFS 4 mo	CTH only as second line treatment. No information on how many patients received CTH. No significant difference was found in mPFS between CTH and everolimus + ET or ET only.
[40]	2022	78, of which 32 received CTH	Retrospective cohort study	1st line palbociclib (n = 55) with: AI n = 18 fulvestrant n = 18 2nd line palbociclib (n = 23) with: fulvestrant n = 20	The study lacks information on specific CTH regimens.	Following progression on 1st line palbociclib: mTTF 6 mo CBR 50% following progression on 2nd line palbociclib: mTTF 8 mo CBR 50%	The majority of patients received CTH subsequent to CDK4/6i.
[41]	2019	136, of which 14 received CTH	Retrospective cohort study	1st line palbociclib (n = 81) with: letrozole n = 66 fulvestrant n = 11 2nd line palbociclib (n = 55) with: letrozole n = 16 fulvestrant n = 15	The study lacks information on specific CTH regimens.	Following progression on 1st line palbociclib: mTTF 4.1 mo following progression on 2nd line palbociclib: mTTF 2.6 mo	No specific comparison between different treatments following CDK4/6i.

Abbreviations: AI, aromatase inhibitors; CBR, clinical benefit rate; CDK4/6i, cyclin-dependent kinase 4 and 6 inhibitors; CTH, chemotherapy; ET, endocrine therapy; mo, months; mOS, median/overall survival; m/rw/PFS, median/real-world/progression-free survival; mTTF, time to treatment failure; N, number of patients in the study; n, number of cases; ORR, objective response rate. Ref, reference; RWD, real-world data; SG, sacituzumab govitecan.

## Data Availability

For access the data, interested parties can contact the corresponding author upon request.
